# Evaluation of *Acinetobacter baumannii*, *Klebsiella pneumoniae,* and *Staphylococcus aureus* respiratory tract superinfections among patients with COVID-19 at a tertiary-care hospital in Tehran, Iran

**DOI:** 10.1186/s40001-023-01303-3

**Published:** 2023-09-02

**Authors:** Maryam Mobarak-Qamsari, Bita Jenaghi, Leyla Sahebi, Mahsa Norouzi-Shadehi, Mohammad-Reza Salehi, Abbas Shakoori-Farahani, Hoda Khoshnevis, Alireza Abdollahi, Mohammad-Mehdi Feizabadi

**Affiliations:** 1 Department of Microbiology, School of Medicine, Tehran University of Medical Sciences, Tehran, Iran; 2 Family Health Research Institute. Maternal, Fetal, and Neonatal Research Center, Tehran University of Medical Sciences, Tehran, Iran; 3 Department of Infectious Disease, School of Medicine, Imam Khomeini Hospital Complex, Tehran University of Medical Sciences, Tehran, Iran; 4 Department of Infectious Diseases, School of Medicine, Imam Khomeini Hospital, Tehran University of Medical Sciences, Tehran, Iran; 5 Department of Medical Genetics, School of Medicine, Imam Khomeini Hospital, Tehran University of Medical Sciences, Tehran, Iran; 6 Imam Khomeini Hospital Complex, School of Medicine, Imam Khomeini Hospital, Tehran University of Medical Sciences, Tehran, Iran; 7 Department of Pathology, School of Medicine, Imam Khomeini Hospital, Tehran University of Medical Sciences, Tehran, Iran; 8 Thorax Research Center, Imam Khomeini Hospital Complex., Tehran University of Medical Sciences, Tehran, Iran

**Keywords:** Healthcare-associated infections, Superinfection, COVID-19, Antibiotic resistance, Respiratory infections

## Abstract

**Background:**

The emergence of healthcare-associated infections (HAIs) or superinfections in COVID-19 patients has resulted in poor prognosis and increased mortality.

**Methods:**

In a cross-sectional study, 101 respiratory samples were collected from ICU-admitted COVID-19 patients. The HAI rate, demographics, and antibiotic resistance were assessed.

**Results:**

The HAI rate was 83.16% (76.62% bacterial and 6.54% fungal). The prevalence of 3 major HAI-causing organisms included *Klebsiella pneumoniae* (41.5%), *Acinetobacter baumannii* (20.8%), and *Staphylococcus aureus* (4.9%). Mortality and intubation ventilation proportions of 90% (*p* = 0.027) and 92.2% (*p* = 0.02) were significant among patients with superinfection, respectively. Multiple logistic regression analysis showed SpO_2_ pressure (odds ratio 0.842; 95% CI 0.750–0.945; *p* = 0.004) as a predictive factor in the association between antibiotic usage and mortality. More than 50% of patients received carbapenems. The resistance rates to at least one antibiotic of third-generation cephalosporins, aminoglycosides, quinolones/fluoroquinolones, tetracyclines, and β-lactam inhibitors were 95.2%, 95.2%, 90%, 57.1%, and 100% among *A. baumannii* isolates and 71.4%, 55%, 69%, 61.9%, and 59.5% among *K. pneumoniae* isolates, respectively. A proportion of 60% was recorded for methicillin-resistant *S. aureus* isolates.

**Conclusion:**

As a result, antibiotic treatment should be administered following the microbial resistance profile. Contact isolation and infection control measures should be implemented as needed.

## Background

**Fig. 1:**
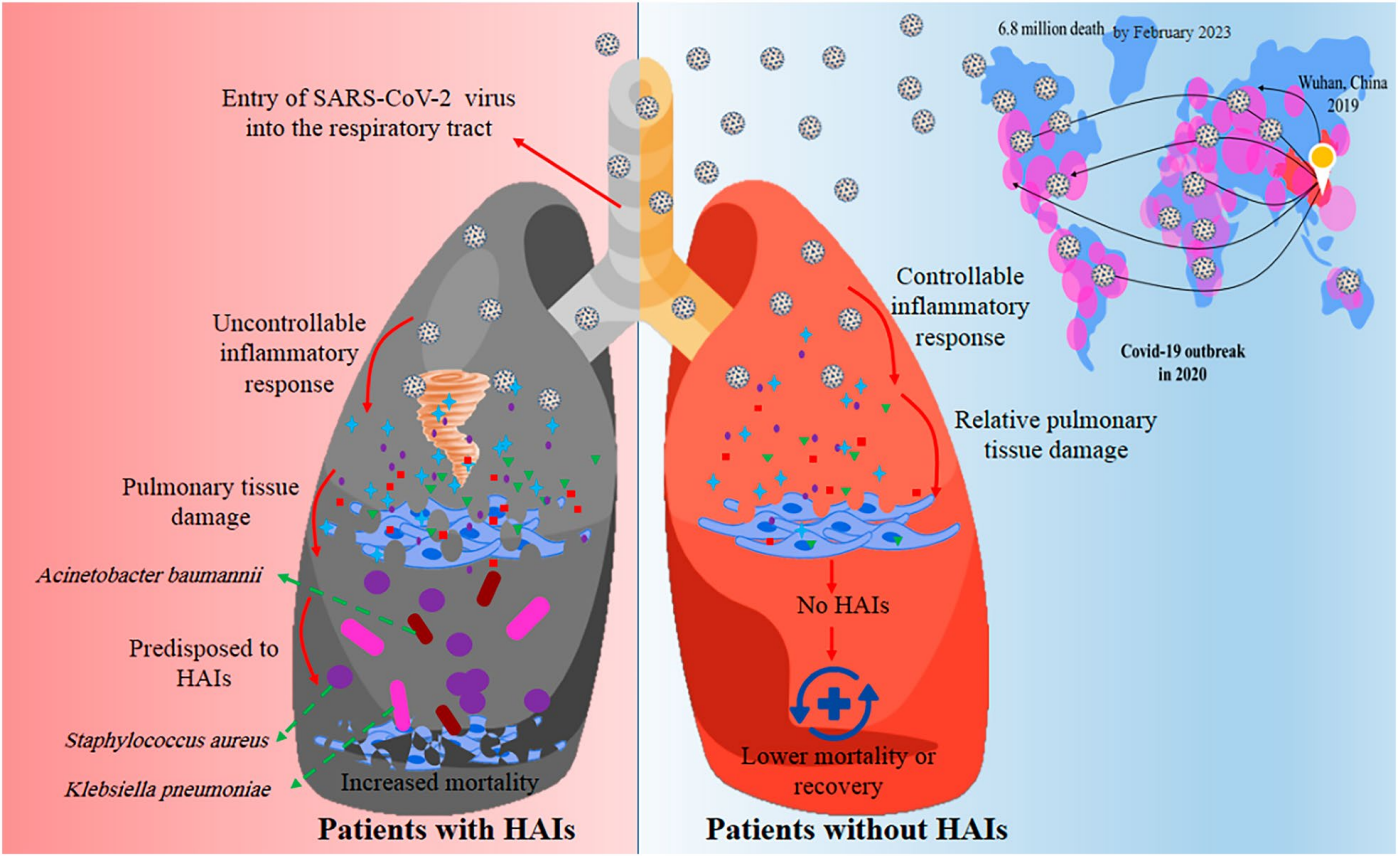
The schematic view of different prognoses of the SARS-CoV-2 virus in predisposing to HAIs

SARS-CoV-2, the newest and seventh member of the Coronaviridae family, is the causative agent of severe respiratory illness in humans, similar to SARS-CoV and MERS-CoV [1], which was discovered in Wuhan’s Hubei Province in China at the end of 2019 and was designated coronavirus disease 2019 (COVID-19) by the WHO on February 11, 2020 [2]. Since its first appearance, the COVID-19 virus has infected more than 753 million individuals worldwide, resulting in over 6.8 million fatalities by February 2023 [3]. This global pandemic has impacted individuals of all ages, causing various clinical symptoms ranging from the common cold to fatal pneumonia [4]. Risk factors and severe clinical COVID-19 cases are usually seen in elderly and immunocompromised individuals who suffer from various concomitant problems, such as cardiovascular diseases, diabetes, hypertension, and chronic obstructive pulmonary disease [5]. SARS-CoV-2 virus can predispose immunocompromised hosts to various bacterial infections by exaggerated production of inflammatory cytokines causing uncontrollable immune responses, multiple organ damage, and severe pneumonia [6]. The lung histopathologic findings reveal the role of healthcare-associated infections (HAIs) causing pneumonia in tissue damage, which can accompanied by increased mortality [7] (Fig. 1).

HAIs are defined as infections that manifest in hospitalized patients at least 48 h after hospital admission and are classified according to the Centers for Disease Control and Prevention/National Healthcare Safety Network (CDC/NHSN) criteria [8]. *K. pneumoniae*, *A. baumannii*, and *S. aureus* isolates are three prevalent microorganisms causing HAI. These organisms are the leading cause of multidrug-resistant nosocomial pathogens, the so-called *Enterococcus faecium*, *S. aureus*, *K. pneumoniae*, *A. baumannii*, *Pseudomonas aeruginosa*, and *Enterobacter* spp. (ESKAPE) group, which account for rising annual healthcare costs and mortality [9].

Carbapenem-resistant *K. pneumoniae* (CRKP) and *A. baumannii* (CRAB) isolates contributed to a significant death rate among critically ill COVID-19 patients with a prolonged hospitalization history [10, 11]. In addition*, S. aureus* isolates are the second leading source of up to 11.8% of HAIs in the United States. They are responsible for both community and HAIs isolated from the lower respiratory tract of COVID-19 patients receiving ventilation [12] (Fig. 1). Given the apparent complication of MRSA in causing ventilator-acquired superinfections in previous SARS-CoV and influenza outbreaks [13], the prevalence of superinfection caused by this organism should be considered in the current pandemic to account for therapeutic targets against this infection [14].

According to the superinfection rate up to 70.6% [15], there has also been a 46.8% increase trend in inappropriate antibiotic treatment among COVID-19 patients with superinfection [16].

This study aimed to assess the prevalence of healthcare-associated respiratory infections or superinfections, antibiotic resistance profiles against the three HAIs mentioned above, and compliance with clinical outcomes among COVID-19 patients admitted to the ICUs of a major hospital in Tehran, Iran.

## Methods

### Ethics statement

The current study was reviewed and approved by the institutional ethics committee of Tehran University of Medical Sciences with an approved ID: IR.TUMS.MEDICINE.REC.1399.1092. Informed consent was obtained from patients or the relatives of unconscious patients. Additionally, due to the probable inconvenience for critical COVID-19 patients and the risk of virus transmission during sampling, we used respiratory samples taken routinely by nursing personnel who were transferred to the microbiology laboratory.

### Study design and sample collection

In a cross-sectional study between March 2021 and July 2021, 101 single and nonduplicate respiratory adult patient samples, including endotracheal aspirate (ETA), bronchoalveolar lavage, and sputum, were collected from confirmed critically ill SARS-CoV-2 patients admitted to Imam Khomeini Hospital Complex, a referral teaching hospital of Tehran University of Medical Sciences. The current study was conducted according to guidelines by the Helsinki Declaration per Strengthening the Reporting of Observational Studies in Epidemiology (STROBE) guidelines, [17], and the collection of 101 respiratory patient samples was based on a pilot study on 20 ICU patients with COVID-19, which estimated the prevalence of nosocomial infections to be 77%, with a type I error of 5% and a power of 80%. The population consisted of all ICU patients with SARS-CoV-2, excluding pregnant women and children.

### Data collection

Data were gathered from electronic medical and nursing records. These included patient demographics, clinical SARS-CoV-2 symptoms, baseline comorbidities, average length of hospital stay, microbial analysis, radiographic findings of lung involvement, SpO_2_ pressure, need for mechanical ventilation, prescribed antibiotic therapy and final disposition (discharged alive or expired). In this study, the severity of coronavirus disease in all confirmed COVID-19 patients was evaluated based on criteria including the need for intubation, respiratory rate > 30 per minute, lung involvement > 50%, and SpO_2_ pressure, which was divided into four categories [mild (SpO_2_ ≥ 93), moderate (90 ≥ SpO_2_ ≥ 92), severe (88.1 ≥ SpO_2_ ≥ 89.9), and critical (SpO_2_ ≤ 88)].

### Reverse transcription-quantitative polymerase chain reaction (RT-qPCR) for detection of confirmed SARS-CoV-2 patients

Nasopharyngeal samples of suspected SARS-CoV-2 patients were tested by multiple one-step quantitative RT-PCR approaches. This experiment was carried out by targeting the E-gene (FAM) and S-gene (ROX) as screening values and detecting the RNaseP (HEX) gene of coronavirus as a confirmatory agent using the Covitech multiplex qPCR kit (ACECR, Iran) according to the manufacturer's instructions.

### Detection of bacterial and fungal superinfection

Respiratory specimens were collected after 48 h of admission and transported to the microbiology laboratory to detect bacterial and fungal infection types by culturing the samples on standard culture media such as blood agar, chocolate agar, and Sabouraud dextrose agar. The generated colonies were then subcultured on several differential culture media, such as MacConkey Agar, Mannitol Salt Agar (MSA), and CHROM Agar Candida media, with incubation at 35–37 °C for 18–24 h. The resulting colonies were validated by Gram staining and a series of standard biochemical and diagnostic tests, such as catalase, urease, Simmon’s citrate, oxidase, triple sugar iron (TSI) agar, sulfide indole motility (SIM), methyl red/Voges–Proskauer (MR/VP), DNase, and lysin decarboxylase. A quantitative culture-based method was also performed to discriminate true infection from potential contamination. A colony-forming unit load of at least 10^4^ per positive culture was considered a true pathogen [18, 19].

To identify the suspected *A. baumannii* colonies, the 16S-23SrRNA gene intergenic spacer (ITS) region and *bla*_*OXA-51-like*_ carbapenemase gene were amplified, as described previously [20]. In addition, for confirmation of the *Candida albicans* complex colonies, the *hwp1* gene was also amplified as described previously [21].

### Antibacterial susceptibility determination

Antimicrobial susceptibility testing was carried out by the Kirby–Bauer disk diffusion method and broth microdilution approaches [22]. The Kirby–Bauer disk diffusion results were interpreted by measuring an inhibition zone around every antibiotic disk in Muller–Hinton agar (MHA) media containing each isolate and categorizing them as susceptible (S), intermediate (I), or resistant (R) according to the zone diameter breakpoint recommended by CLSI guidelines [22]. The antibiotic disks (Rosco, Taastrup, Denmark) used against each bacterial group consisted of the following:

### Antibiotic disks were used against *K. pneumoniae* isolates

Cefazolin (CZ, 30 µg), cefepime (CPM, 30 μg), cefotaxime (CTX, 30 µg), ceftriaxone (CRO, 30 µg), ceftazidime (CAZ, 30 μg), aztreonam (ATM, 30 µg), imipenem (IPM, 10 μg), meropenem (MER, 10 μg), gentamycin (GM, 10 µg), tobramycin (TM, 10 µg), amikacin (AN, 30 µg), tetracycline (TE, 30 µg), doxycycline (DO, 30 µg), levofloxacin (LVX, 5 μg), ciprofloxacin (CP, 5 µg), ofloxacin (OFL, 5 µg), trimethoprim–sulfamethoxazole (SXT, 1.25/23.75 µg), chloramphenicol (C, 30 µg), piperacillin–tazobactam (TZP, 100/10 µg), and ampicillin–sulbactam (SAM, 10/10 µg).

### Antibiotic disks were used against *A. baumannii* isolates

Cefepime (CPM, 30 μg), cefotaxime (CTX, 30 µg), ceftriaxone (CRO, 30 µg), ceftazidime (CAZ, 30 μg), imipenem (IPM, 10 μg), meropenem (MER, 10 μg), gentamycin (GM, 10 µg), tobramycin (TM, 10 µg), amikacin (AN, 30 µg), doxycycline (DO, 30 µg), levofloxacin (LVX, 5 μg), ciprofloxacin (CP, 5 µg), trimethoprim–sulfamethoxazole (SXT, 1.25/23.75 µg), piperacillin–tazobactam (TZP, 100/10 µg), and ampicillin–sulbactam (SAM, 10/10 µg).

### Antibiotic disks were used against *S. aureus* isolates

Gentamycin (GM, 10 µg), amikacin (AN, 30 µg), tetracycline (TE, 30 µg), doxycycline (DO, 30 µg), levofloxacin (LVX, 5 μg), ciprofloxacin (CP, 5 µg), ofloxacin (OFL, 5 µg), trimethoprim–sulfamethoxazole (SXT, 1.25/23.75 µg), chloramphenicol (C, 30 µg), penicillin (P, 10 U), cefoxitin (FOX, 30 μg), azithromycin (AZM, 15 μg), clindamycin (CD, 2 µg), rifampin (RA, 5 µg), and linezolid (LZD, 30 µg).

### The broth microdilution assay

The broth microdilution (BMD) assay was performed by the minimum inhibitory concentration (MIC) method to evaluate the antimicrobial effect of colistin (COL) against *A. baumannii* and *K. pneumoniae* isolates and vancomycin (VAN) and oxacillin (OXA) against *S. aureus* isolates. The standard MIC breakpoints of COL against *A. baumannii* and *Enterobacterales* (MIC ≤ 2 μg/ml: intermediate and MIC ≥ 4 μg/ml: resistant) were employed. Additionally, the MIC breakpoints of VAN (MIC ≤ 2 µg/ml: susceptible, 4 ≤ MIC ≤ 8: intermediate, and MIC ≥ 16: resistant) and OXA (MIC ≥ 4 µg/ml: resistant and MIC ≤ 2 µg/ml: susceptible) against *S. aureus* isolates were used [22]. *Escherichia coli* (ATCC 25922), *P. aeruginosa* (ATCC 27853), *S. aureus* (ATCC 25923), and *S. aureus* (ATCC 700699) were treated as controls. Detection of MRSA isolates was further confirmed by the amplification of the *mec-A* gene by PCR [23].

### Determination of MDR and ESBL-producing isolates

The prevalence of MDR isolates was assessed. MDR isolates are categorized as resistant or nonsusceptible to at least one antimicrobial agent among three or more antibiotic classes [24]. Additionally, the combination disk technique was used to examine the extended-spectrum beta-lactamase (ESBL)-producing isolates. Ceftazidime (CAZ, 30 g) and ceftazidime–clavulanate (CAV, 30/10 g, Mast), cefotaxime (CTX, 30 g), and cefotaxime–clavulanate (CV, 30/10 g, Mast) were used on Muller–Hinton agar inoculated by suspected ESBL-producing isolates [25]. The positive ESBL-producing *K. pneumoniae* isolates were verified by a ≥ 5 mm increase in inhibition zone diameter surrounding cefotaxime–clavulanate and/or ceftazidime–clavulanate compared to the diameter inhibition zone of ceftazidime and cefotaxime separately [26].

### Statistical analysis

Student’s *t* test was used to find the mean (SD) of continuous variables through a comparative analysis. The Chi-square test or Fisher's exact Chi-square test was employed to compare categorical variables. Univariate and multiple logistic regression analyses were conducted to determine the parameters independently associated with antibiotic treatment and mortality in COVID-19 patients with and without HAIs. Initially, univariate logistic regression was conducted by considering all causes of mortality. The predictive variables with a *p* value ≤ 0.2 were selected and introduced into the multiple logistic regression model to control the effect of confounding variables. *P* values less than 0.05 were considered statistically significant. All statistical analyses were performed with IBM SPSS Statistics 26.0.

## Results

### Clinical and demographic features

HAI was diagnosed in 89% (84/101) of confirmed COVID-19 patients. Patients with HAI were older on average, with a mean age of 61 (SD 12.45; IQR 17) years; more than half (49/84) were men (58.3%). Almost all clinical symptoms and severity of COVID-19 increased in individuals with HAI compared to those without HAI. As a result, lung involvement of 50% was only recorded in patients with HAI. The median length of hospital stay duration was 18 (IQR 17) days (*p* = 0.184). The proportion of in-hospital mortality (90%, 63/70) (*p* = 0.027) and the need for intubation ventilation (92.2%, 59/64) (*p* = 0.02) were significantly higher in patients with HAI. Almost 84.3% (59/70) of expired patients were under mechanical ventilation (*p* = 0.000). Hypertension, diabetes mellitus, immunodeficiency, and coronary heart disease were more prevalent in patients with HAI, as shown in Table 1.

**Table 1:** The main characteristics of ICU-admitted patients with SARS-COV-2

	Patients with SARS-COV-2; *n* (%)			
	All patients (101)*n* (%)	With HAI 84 (89%)	Without HAI 12 (11%)	*P* value
Age, mean (SD)	60.72 (12.81)	61.13 (12.45)	58.56 (14.81)	0.465^a^
Gender				
Male	58 (57.4)	49 (58.3)	9 (52.9)	0.682^b^
Female	43 (42.6)	35 (41.7)	8 (47.1)	
Comorbidities				
Hypertension	46 (46.9)	37 (80.4)	9 (19.6)	0.415^b^
Diabetes mellitus	20 (20.4)	17 (85.0)	3 (15.0)	1.0^c^
Coronary heart disease	8 (8.2)	6 (75.0)	2 (25.0)	0.613^c^
Immunocompromising diseases^1^	9 (9.2)	8 (88.8)	1 (11.1)	1.0^c^
Kidney disease^2^	3 (3.12)	2 (66.7)	1 (33.3)	1.0^c^
Asthma	5 (5.1)	4 (80.0)	1 (20.0)	1.0^c^
Other underlying disease^3^	4 (4.1)	2 (50.0)	2 (50.0)	0.063^c^
COVID symptoms				
Shortness of breath	70 (72.9)	61 (87.1)	9 (12.9)	0.52^c^
Lethargy and fatigue	61 (63.5)	50 (82.0)	11 (18.0)	0.21^b^
Fever	54 (56.3)	48 (88.9)	6 (11.1)	0.247^b^
Myalgia	54 (56.30)	37 (88.1)	5 (11.9)	0.512^b^
Sputum cough	51 (53.1)	22 (78.6)	6 (21.4)	0.339^b^
Chills	30 (31.3)	26 (78.6)	6 (21.4)	1.0^b^
Chest pain	14 (14.6)	4 (100.0)	0 (0.0)	0.21^c^
Headache	13 (13.5)	9 (69.2)	4 (30.8)	0.094^b^
Diarrhea and nausea	14 (14.14)	12 (85.7)	2 (14.3)	1.0^c^
Other symptoms ^4^	20 (21.05)	16 (80.0)	4 (20.0)	0.463
Severity of COVID				
Need to intubation	64 (63.4)	59 (92.2)	5 (7.8)	0.02
Respiration rate	47 (48.45)	38 (80.9)	9 (19.1)	0.495^b^
Mild	17 (.17.17.)	15 (88.2)	2 (11.8)	0.139^d^
Moderate	22 (22.22)	18 (81.8)	4 (18.2)	
Sever	2 (.2.)	2 (100)	0 (0)	
Critical	58 (.58.58.)	48 (82.8)	10 (17.2)	
Lung involvement ≥ 50%	7 (7)	7 (100.0)	0 (0)	1.0^c^
Outcome of inpatient				
Expired	70 (69.3)	63 (90.0)	7 (10.0)	0.027^b^
Length of hospital stay, mean (IQR)	18 (17)	18 (14)	10 (28.5)	0.184
Treatments				
Antibiotics^5^	83 (86.45)	71 (85.5)	12 (14.5)	0.93^b^
Antifungals^6^	12 (12.5)	11 (91.7)	1 (8.3)	0.512^b^
Remdesivir	50 (52)	43 (86)	7 (14)	0.866^b^
Corticosteroids^7^	50 (52)	42 (84)	8 (16.0)	0.682^b^
Vitamins/complements^8^	6 (6.25)	6 (100)	0 (0)	0.558^b^
Other drugs^9^	46 (48)	39 (84.8)	7 (15.2)	0.866^b^

^a^*P* value by independent sample *T* test

^b^*P* value by Chi-square test

^c^Fisher’s exact *P* value by Chi-square test

^d^Linear by linear *P* value by Chi-square test

^1^Immune deficiency: breast cancer, colon cancer, intestinal cancer, liver transplant, kidney transplant, brain tumor, and HIV

^2^Kidney disease: benign bladder tumor, kidney stone, and lupus nephritis

^3^Other underlying diseases: epilepsy, Parkinson, depression, and addiction

^4^Other symptoms: sweating, bloody sputum, runny nose, sore throat, anorexia, anosmia, loss of taste, stomachache, weight loss, dizziness, dry cough, whooping cough, and loss of consciousness

^5^Antibiotics: colistin, imipenem, meropenem, levofloxacin, ciprofloxacin, erythromycin, linezolid, vancomycin, cefepime, cefazolin, metronidazole, piperacillin–tazobactam, cotrimoxazole, amikacin, gentamycin, ceftriaxone, azithromycin, clindamycin, doxycycline, and tigecycline

^6^Antifungal: caspofungin, Amphotericin B, and fluconazole

^7^Corticosteroids: prednisolone, dexamethasone, and hydroxychloroquine

^8^Vitamins/complements: zinc, vitamin D, and vitamin C

^9^Other drugs: heparin, melatonin, aspirin, diphenhydramine, acetaminophen, and naproxen

### Microbial and fungal isolates causing HAI

Bacterial and fungal superinfections accounted for 82.17% and 5.94%, respectively. Approximately 65.3% of samples (66/101) were monomicrobial, 17.8% (18/101) were multimicrobial, and 1.98% (2/101) included bacterial and fungal infections and were considered mixed infections, while 11.88% (12/101) of patient samples had no culturable infections. The isolated microorganisms were *K. pneumoniae* (41.5%, 42/101), *A. baumannii* (20.8%, 21/101), *S. aureus* (4.9%, 5/101), *E. coli* (3.9%, 4/101), *P. aeruginosa* (3.9%, 4/101), *S. epidermidis* (2.9%, 3/101), *Citrobacter* spp. (1.9, 2/101), *Enterobacter* spp. (0.9%, 1/101), *Stenotrophomonas* spp. (0.9%, 1/101) and *Candida albicans complex* (5.94%, 6/101). One expired patient with lung involvement ≥ 80% was diagnosed with bacterial and fungal superinfection.

### Medications and HAI

Antibiotics, antivirals (remdesivir), antifungals, corticosteroids, and other associated drugs were prescribed more frequently in COVID-19 patients with HAI (Table 1). More than 85.5% of the patients with HAI received at least one antibiotic. While more than 55% of all patients were under treatment with carbapenems (IPM and MER), almost 86% of patients who received carbapenems had HAI. Meropenem (53.7%, 44/50), COL (37.8%, 31/33), and VAN (39%, 32/37) were the most commonly used antibiotics in patients with HAI. Except for one discharged patient, no history of antibiotic therapy was recorded. Univariate logistic regression revealed that age (men) (OR, 1.056; CI 95%, 1.017–1.097), (*p* = 0.004), total HAI (OR, 3.789; CI 95%, 1.216–11.810), (*p* = 0.022), SpO_2_ pressure (OR, 0.864; CI 95%, 0.786- 0.949), (*p* = 0.002), and antibiotic use (OR, 3.471; CI 95%, 1.240–9.714), (*p* = 0.018) could be independent factors associated with in-hospital mortality. The multiple regression analysis and SpO_2_ pressure (OR, 0.842; CI 95%, 0.750–0.945), (*p* = 0.004) were ascertained as the final predictive factors in the correlation between antibiotic use and in-hospital mortality, as shown in Table 2.

**Table 2:** Univariate and multiple logistic regression analysis of the association between antibiotic use and outcome

	Odds ratio (95%CI)	*P* value
Univariate analyses		
Age	1.056 (1.017–1.097)	0.004
Genus; males	1.351 (0.543–3.365)	0.518
Having HAI	3.789 (1.216–11.810)	0.022
SpO_2_ pressure	0.864 (0.786–0.949)	0.002
Taking corticosteroids	0.833 (0.332–2.084)	0.695
Taking antifungals	1.917 (.390–9.422)	0.423
Taking antibiotics	3.471 (1.240–9.714)	0.018
Taking vitamins	1.846 (0.205–16.620)	0.584
Having comorbidities	1.60 (0.645–3.972)	0.311
Multiple analyses		
Age; males	1.037 (0.993–1.084)	0.101
Having HAI	1.569 (0.087–28.179)	0.760
SpO_2_ pressure	0.842 (0.750–0.945)	0.002
Taking antibiotics	3.394 (0.227–50.811)	0.376

### Antibacterial susceptibility profile

**Fig. 2:**
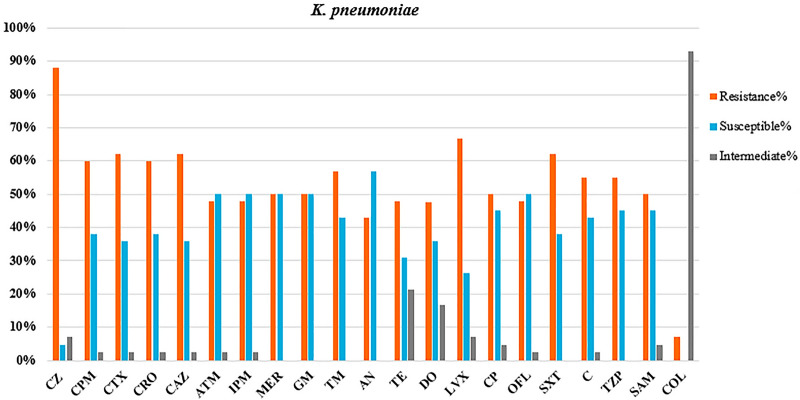
The antimicrobial susceptibility testing against HAI-causing *K. pneumoniae* isolates. CZ cefazolin, CPM cefepime, CTX cefotaxime, CRO ceftriaxone, CAZ ceftazidime, ATM aztreonam, IPM imipenem, MER meropenem, GM gentamycin, TM tobramycin, AN amikacin, TE tetracycline, DO doxycycline, LVX levofloxacin, CP ciprofloxacin, OFL ofloxacin, SXT trimethoprim–sulfamethoxazole, C chloramphenicol, TZP piperacillin–tazobactam, SAM ampicillin–sulbactam, COL colistin

**Fig. 3:**
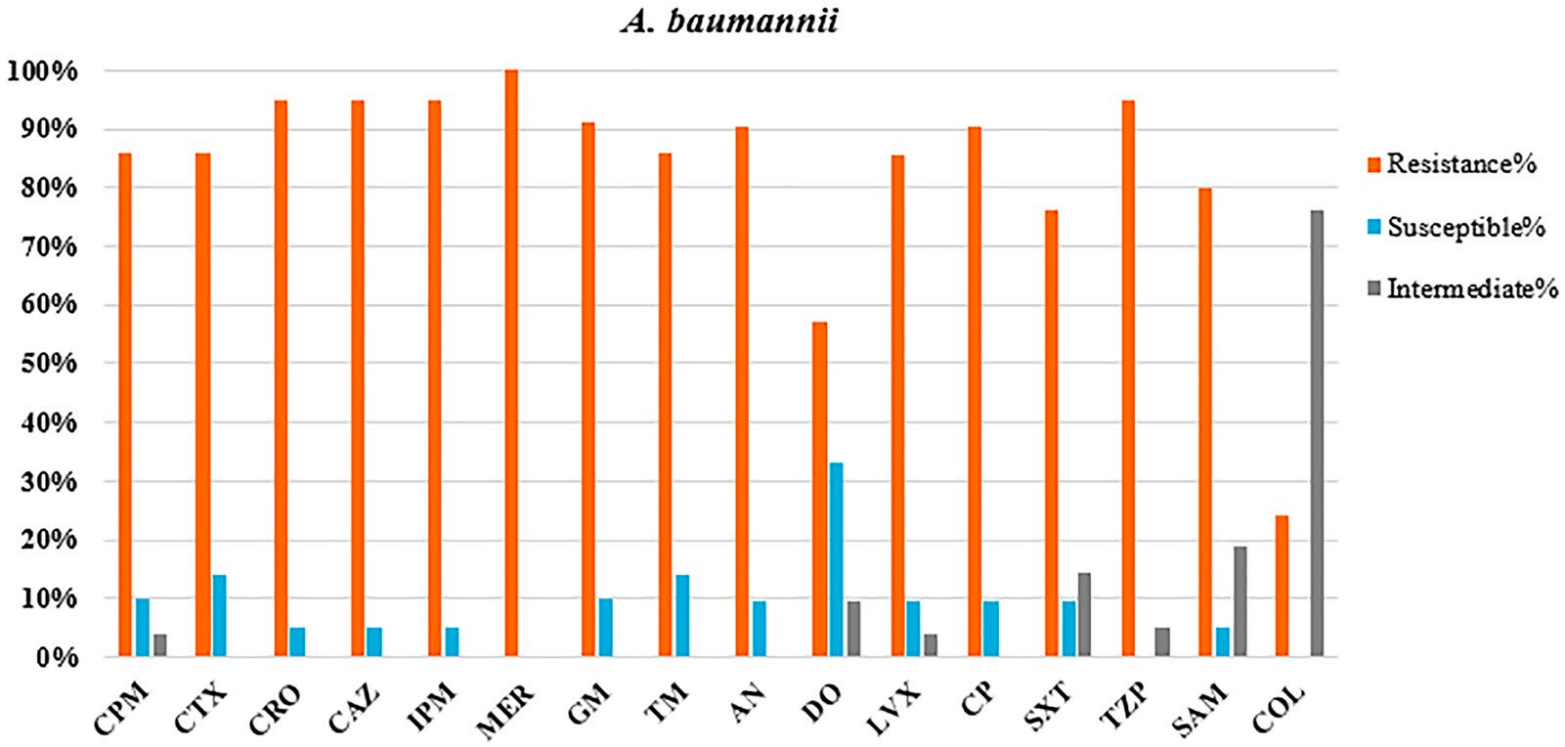
Antimicrobial susceptibility testing against HAI-causing *A. baumannii* isolates. CPM cefepime, CTX cefotaxime, CRO ceftriaxone, CAZ ceftazidime, IPM imipenem, MER meropenem, GM gentamycin, TM tobramycin, AN amikacin, DO doxycycline, LVX levofloxacin, CP ciprofloxacin, SXT trimethoprim–sulfamethoxazole, TZP piperacillin–tazobactam, SAM ampicillin–sulbactam, COL colistin

**Fig. 4:**
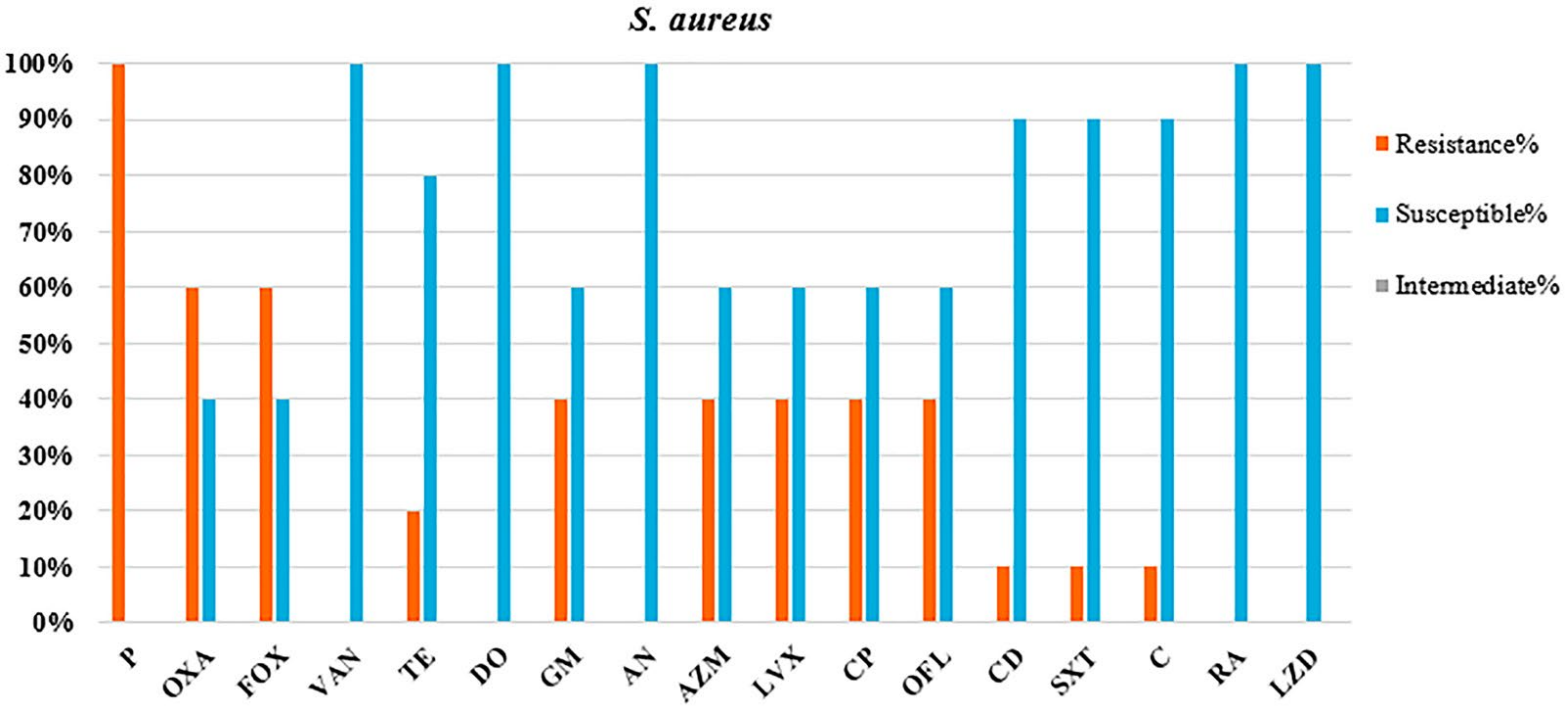
Antimicrobial susceptibility testing against HAI-causing *S. aureus* isolates. P penicillin, OXA oxacillin, FOX cefoxitin, VAN vancomycin, TE tetracycline, DO doxycycline, GM gentamycin, AN amikacin, AZM azithromycin, LVX levofloxacin, CP ciprofloxacin, OFL ofloxacin, CD clindamycin, SXT trimethoprim–sulfamethoxazole, C chloramphenicol, RA rifampin, LZD linezolid

The results of antibacterial susceptibility testing, as shown in Figs. 2, 3, 4, demonstrated a dramatic increase in antibiotic resistance among *K. pneumoniae* and *A. baumannii* isolates. CRAB and CRKP proportions of 100% and 50% were detected against at least one antibiotic of carbapenems (MER and/or IPM). Approximately 24% of *A. baumannii* and 7.14% of *K. pneumoniae* isolates were resistant to COL. The resistance proportion to at least one antibiotic of third-generation cephalosporins (CTX, CRO, and CAZ), aminoglycosides (AN, TM, and GM), quinolones/fluoroquinolones (LVX, CP, and OFL), tetracyclines (TE and DO), and β-lactam inhibitors (TZP and SAM) was 95.2%, 95.2%, 90%, 57.1%, and 100% among MDR *A. baumannii* isolates and 71.4%, 55%, 69%, 61.9%, and 59.5% among *K. pneumoniae* isolates, respectively. In addition, resistance to SXT increased against two Gram-negative bacteria.

Although all five *S. aureus* isolates in our study (100%) were susceptible to VAN (MIC ≤ 2 µg/ml), AN, LZD, RA, and DO, all were resistant to P. Of the five isolates of *S. aureus*, three (60.0%) were resistant to FOX and OXA (MIC ≥ 4 µg/ml), suggesting that they were MRSA and were eventually confirmed by amplification of the *mec-A* gene. The MRSA isolates (*n* = 3/5) were resistant to at least three (16.6%) and a maximum of 11 (61.1%) antibiotics, while MSSA isolates (2/5) were resistant to at least one (5.6%, 1/18) and a maximum of six (33.3%, 6/18) antibiotics. Resistance proportions of 66.6% to GM and 33.3% to TE, AZM, LVX, CP, OFL, CD, and SXT were obtained among MRSA isolates. Meanwhile, the MSSA isolates showed a resistance proportion of 50% (1/2) to AZM, LVX, CP, OFL, and C. Two (66.6%, 2/3) patients infected with MRSA isolates had critical symptoms and died.

### The MDR and ESBL-producing isolates

The MDR proportion was 100% and 71.4% among *A. baumannii* and *K. pneumoniae* isolates, respectively. Interestingly, 82.71% (18/21) of MDR *A. baumannii* isolates and 86.2% (25/29) of MDR *K. pneumoniae* isolates led to 100% mortality in patients with HAI. The ESBL-producing *K. pneumoniae* isolates were 24% (10/42). Additionally, all ESBL-producing *K. pneumoniae* isolates were MDR.

## Discussion

This study detected a high HAI proportion of 83.2% among ICU-admitted COVID-19 patients. Most patients with HAI were critically ill. Additionally, we were in lacked access to microbiological data during the first admission. Thus, some might have been admitted with coinfections that were classified as superinfections. Furthermore, the length of stay in our study was relatively long (median = 18 days). In numerous other studies, the length of hospital stay was prolonged for patients with various superinfections and was accompanied by increased mortality [27–29].

Approximately 92% of patients with HAI were intubated, and of those, 80% had at least one underlying disease, which led to a mortality rate of 90%. Similarly, in some investigations, hypertension, diabetes, and coronary heart disease were found to be most common among patients suffering from comorbidities and HAI [29, 30]. In addition, 84% of patients with HAI received immunosuppressive therapies more frequently, primarily because of multiple organ damage due to hyperinflammatory immune responses in critically ill COVID-19 patients [31]. These patients may have been infected by various bacterial, fungal, and viral superinfections that exacerbated the severity of their symptoms [32]. These factors may be justifiable for our high incidence of HAI.

In our study, antibiotic administration was more frequent among patients with HAI than those without it, which had a significant association with mortality. In addition, HAI-causing *A. baumannii* and *K. pneumoniae* isolates exhibited high resistance to many antibiotics. Similarly, in the Floridia et al*.* study, the most frequent pathogens causing infections were *Enterobacterales,* mainly *K. pneumoniae*, followed by *A. baumannii* and *S. aureus* isolates. In addition, a higher rate of antibiotic resistance was exhibited by these organisms during the first and second waves of COVID-19 than during a prior pandemic [33]. In one study by Kariyawasam et al., MDR isolates of *K pneumoniae* (*n* = 274), *A. baumannii* (*n* = 218), *P. aeruginosa* (*n* = 203), and MRSA (*n* = 132) were more frequent than the others [34]. Similar to the Mdrzycka et al. study, the highest prevalence of CRKP was up to 53%, mainly occurring in elderly and male SARS-CoV-2-infected individuals [35].

In agreement with our findings, Tadesse et al. stated the presence of carbapenemase and many antimicrobial resistance (AMR)-encoding genes, such as ESBLs, on mobile genetic elements to facilitate the accumulation and spread of AMR genes. This emphasizes why most carbapenem-resistant Gram-negative bacteria, such as CRKP and CRAB isolates, are primarily resistant to other antibiotic classes, such as aminoglycosides, fluoroquinolones, and SXT [36]. In our study, all ESBL-producing *K. pneumoniae* isolates were MDR. Mazzariol et al. reported a proportion of 61.5% (8/13) for CRKP and 23.5% (3/13) for ESBL-producing *K. pneumoniae*, which was comparable to our findings [37].

In one study by Pourajam et al., in contrast to our results, the superinfection-causing *K. pneumoniae* isolates showed the highest antibiotic resistance (97.9%) to CAZ, CRO, CPM, SAM, PTZ, and CP. They had increased resistance to LVX (95.8%), MER (95.8%), GM (93.6%), and AN (80.9%). While similar to our results, the resistance rate of *A. baumannii* isolates to COL was 10.6%, the full resistance rate to CAZ, CRO, CPM, TZP, MER, and CP, and 97.2%, 94.3%, and 60% resistance to GM, AN, and SAM were recorded, but no resistance rate to COL was found [38]. In the Bahce et al*.* study, similar to our study, the *A. baumannii* isolates showed full resistance to MEM, TPZ, CIP, LEV, and IMP. They also revealed an increased resistance to AK (63.8%), GN (95.7%), and TM (97.7%). In contrast to our results, the resistance rate of *A. baumannii* isolates to SXT was low (46.8%). Additionally, the resistance rate of *K. pneumoniae* isolates to GN (44.4%), COL (42.9%), MEM (71.4%), TZP (77.8%), CIP (100%), and FEP (77.8%) was reported to be relatively high compared to our results [39].

In a cross-sectional study by Qodrati et al., a low rate of MRSA (37.5%, 216/576) was obtained from clinical specimens one year before the pandemic. The antibiotic resistance against MRSA isolates was greater than that against MSSA isolates and was similar against GM, TE, LVX, SXT, CP, CD, and SXT. Given the full susceptibility of VAN and LZD to all *S. aureus* isolates, the authors considered these drugs as the first treatment priority against MRSA-causing infections [40]. Despite the frequent use of VAN in hospitalized COVID-19 patients, low numbers of MSSA (2/5) and MRSA isolates (3/5) were observed in our study. Additionally, Falcon [41] and Punjabi et al. [13] reported a low incidence of *S. aureus* and culture-based MRSA (5.7%) isolates from respiratory COVID-19 patient samples. In contrast to Qodrati et al., Punjabi et al. believed that continuous VAN treatment might not be more beneficial for superinfections caused by MRSA isolates [13].

Although the prescription of antimicrobial treatment in infections of the ongoing SARS-CoV-2 pandemic is unavoidable, there has been no documented sign of its efficacy in lowering mortality [42]. Some reports have shown widespread antibiotic use challenges in body metabolism and could prevent the body from generating proper antibodies against SARS-CoV-2 infections. This could be one of the main reasons for the high mortality in the ongoing COVID-19 pandemic [43]. The presence of MDR Gram-negative isolates in our investigation might justify the elevated HAI rate and consequent high incidence of superinfected patient mortality.

The current study had certain limitations. First, all analyses were conducted solely on patients with SARS-CoV-2, and the frequency of HAI in hospitalized patients without COVID-19 was not evaluated. Second, the diagnosis of HAI was based on the microbial culture results. We had some constraints in performing the molecular-based methods to detect additional atypical infectious agents. Last, we did not have access to the antibiotic administration data and preliminary microbiological culture results before hospitalization to evaluate the efficacy of antimicrobial regimens on the type and proportion of HAIs among SARS-CoV-2 patients.

## Conclusion

The present study reveals a substantial prevalence of bacterial and fungal superinfections (83.2%) among SARS-CoV-2 ICU-admitted patients, particularly MDR CRKP and CRAB isolates. The superinfections were significantly associated with increased mechanical ventilation (92.2%) and high mortality (90%). To mitigate antimicrobial resistance, patients were treated according to their precise microbial resistance profile using antibiotic regimens based on the clinicians’ recommendations and hospital procedures. Additionally, implementing contact isolation and infection control programs is necessary if needed.

## Data Availability

The datasets generated and analyzed in the current study are available from the corresponding author upon reasonable request.

## Abbreviations

SARS-CoV-2: Severe acute respiratory syndrome coronavirus 2
ICU: Intensive care unit
RT-qPCR: Reverse transcription-quantitative polymerase chain reaction
HAI: Healthcare-associated infections
CLSI: Clinical and Laboratory Standards Institute
CZ: Cefazolin
CPM: Cefepime
CTX: Cefotaxime
CRO: Ceftriaxone
CAZ: Ceftazidime
ATM: Aztreonam
IPM: Imipenem
MER: Meropenem
GM: Gentamycin
TM: Tobramycin
AN: Amikacin
TE: Tetracycline
DO: Doxycycline
LVX: Levofloxacin
CP: Ciprofloxacin
OFL: Ofloxacin
SXT: Trimethoprim–sulfamethoxazole
C: Chloramphenicol
TZP: Piperacillin–tazobactam
SAM: Ampicillin–sulbactam
COL: Colistin
P: Penicillin
OXA: Oxacillin
FOX: Cefoxitin
VAN: Vancomycin
AZM: Azithromycin
CD: Clindamycin
RA: Rifampin
LZD: Linezolid
MICs: Minimum inhibitory concentrations
CDDT: Combination disk diffusion
ATCC: American Type Culture Collection
ESBL: Extended spectrum beta-lactamases
MDR: Multi drug resistance
MRSA: Methicillin-resistant *Staphylococcus aureus*
MSSA: Methicillin-sensitive *Staphylococcus aureus*
